# Check'-ing DLBCL

**DOI:** 10.18632/oncoscience.124

**Published:** 2015-02-08

**Authors:** Itziar Salaverria, Sílvia Beà

**Affiliations:** Hematopathology Unit, Hospital Clínic, Institut d'Investigacions Biomèdiques August Pi i Sunyer (IDIBAPS), Barcelona, Spain

*MYC* is a potent oncogene that encodes a transcription factor which regulates a plethora of target genes related to cell growth and cell cycle. Not surprisingly, a vast majority of human cancers are characterized by high constitutive Myc levels promoting oncogenesis. In particular, in mature B-cell neoplasms Myc overexpression is often associated with an aggressive clinical behavior. Interestingly, a high degree of Myc overexpression can be sensed as an oncogenic stress, and, as a protective mechanism, the cell can experience senescence or apoptosis. Myc not only causes replication stress but also activates the DNA damage response (DDR) through ATM and ATR kinases, which, in response to DNA breaks switch on a transduction pathway that activates p53. This cascade activates Chk1, which, in turn, inactivates Cdc25 phosphatase and other proteins of the replication fork and spindle checkpoint. All these modifications promote G2/M cell cycle arrest, in order to facilitate DNA repair and thus, prevent the cell from a premature and catastrophic mitosis in cells harboring extensive DNA damage.

Most human tumors are characterized by genomic instability, some of its manifestations are lagging chromosomes and chromatin bridges, and multipolar spindles caused by supernumerary centrosomes that cause anomalous chromosome segregation. Genomic instability may be “tolerated” by a low level of checkpoint bypass. In contrast, an extremely high level of genomic instability -due to Chk1 inhibition, for example- can be used as a therapeutic strategy, since tumor cells treated with chemo or radiotherapy along with Chk1 inhibitors are more sensitive than normal cells. This link between Myc and Chk1 was first demonstrated by Höglund et al [1] in 2011 when they treated mouse models of Myc-driven B-cell lymphomas with Chk1 inhibition and they observed marked caspase-dependent apoptosis. They suggested that not only Myc-driven B-cell lymphomas would benefit from this type of therapy, but also neuroblastoma, and breast and lung cancers, all of them characterized by high levels of Myc [1,2]. The rationale behind this observation is that tumors with high levels of Myc become dependent of Chk1 for maintaining genomic integrity, and by adding Chk1 inhibitors to the standard therapy regimens the cells increase its genomic instability to “untolerated” levels and become more sensitive to treatment (Figure 1). This phenomenon is termed “synthetic letal”, and requires deregulation of both genes, Myc and Chk1 simultaneously. Thus, Myc-driven tumors are perfect candidates for Chk1-inhibition as a therapeutic strategy specially cases with mutated p53, proven to be insensitive to other types of therapies. Moreover, Ferrao et al. [3] demonstrated the benefits and efficacy of treating Myc-driven lymphomas with single agent Chk inhibitor and showed that the dual Chk1-Chk2 inhibitor was more powerful in p53 deficient cells than the Chk1 inhibitor alone.

**Figure:**
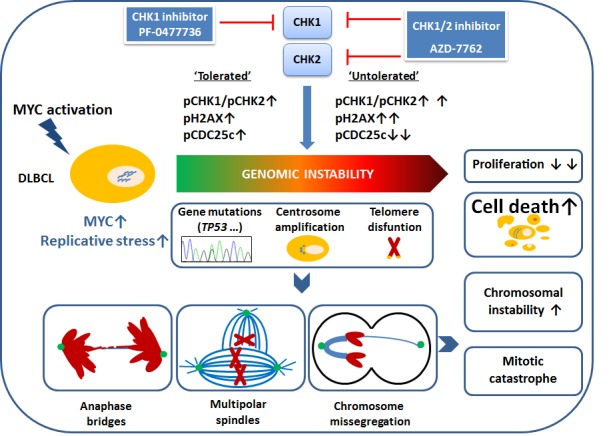
Targeting MYC-induced genomic instability in DLBCL with CHK inhibitors

In a recent issue of Oncotarget, Derenzini et al [4] point to the inhibition of DDR, via Chk inhibition, as a potential therapeutic strategy in diffuse large B-cell lymphoma (DLBCL). Cells with Myc-driven replicative stress and genomic instability after Chk inhibition were driven to mitotic catastrophe due to high levels of DNA damage and with “untolerated” genomic instability that eventually ended in cell death (Figure 1). They studied the expression of Myc, phosporylated Chk1/2 and Cdc25c proteins, and phosporylated histone γH2AX, as a bona fide marker of inherent DNA damage. Both overexpression of Myc and constitutive expression of γH2AX were tightly related and associated with poor outcome of patients after standard chemotherapy regimens. The authors demonstrated a reduction of proliferation through inhibition of the DDR pathway using Chk inhibition. Overall, these results and previous observations suggest that pharmacological inhibition of Chk1 could represent a novel and efficient therapeutic strategy not only for DLBCL with Myc overexpresssion, but also for other malignant B-cell lymphomas characterized by high Myc levels and high degree of genomic instability, like mantle cell lymphomas, even in cases with inactive p53. Interestingly, chromosomal missegregation defects in DLBCL visualized under the microscope are much more frequent in patients with short overall survival and inferior response to therapy [5].

Some concerns before including this strategy in clinical trials are the difficulties in standardizing the quantitative evaluation of MYC expression in tumors. Similarly, it is not clear what could be the most appropriate surrogate marker for genomic instability, γH2AX phosporylation, or directly study the chromosomes or copy number arrays?

Recently, targeted sequencing of key DNA repair genes in DLBCL has found mutations in several genes, including 8% mutations of *CHK2* [6], some of them of germline origin. Occasional mutations and loss of Chk2 protein expression in aggressive lymphomas, such as mantle cell lymphomas, had also been previously associated with high levels of chromosomal instability [7]. Overall, all these studies highlight the importance of the DDR pathway in aggressive lymphoma pathogenesis, but the functional consequences and also their direct link with chromosomal instability have still to be elucidated. Moreover, it should be reasonable to try the strategy of Chk inhibition in combination with chemotherapy in these cases with mutations in DNA repair genes to test if it is still efficient. It should also be taken into consideration that the different genetic background of each tumor may determine the degree of efficacy of Chk inhibitors. In summary, Chk inhibition in MYC-driven tumors should be considered in the current era of molecular targeted therapies and genome/exome sequencing.
